# Resonant Leadership and Proactive Vitality Management in Nurses: The Mediating Role of Post–COVID-19 Organizational Compassion

**DOI:** 10.1097/jnr.0000000000000695

**Published:** 2025-07-08

**Authors:** Nadia Hassan Ali Awad, Nariman Ahmed Mohamed Elbassal, Heba Mohamed Al-anwer Ashour

**Affiliations:** 1 Nursing Administration Department, Faculty of Nursing, Alexandria University, Alexandria, Egypt; 2 Nursing Program, Batterjee Medical College, Jeddah, Saudi Arabia; 3 College of Nursing, King Saud Bin Abdul-Aziz University for Health Sciences, King Abdulaziz Medical City, Jeddah, Saudi Arabia; 4 King Abdullah International Medical Research Center, Jeddah, Saudi Arabia

**Keywords:** COVID-19, resonant leadership, structure equation model, proactive vitality management, nurses, organizational compassion

## Abstract

**Background:**

In the post–COVID-19 era, health care organizations must become more compassionate and focus on empathizing with their employees, noticing change in their behavior through continuous assessment, and responding to their suffering. Thus, health care organization leaders must be more emotionally responsive and provide compassionate support to the nurses through a resonant leadership style. Resonant leaders exhibit passionate insights, are attuned to the emotions of their followers, employ empathy, and effectively manage their feelings to build strong, trustworthy relationships and foster an environment of hope that motivates dedication. Consequently, applying a resonant leadership style in the presence of organizational compassion can enhance nurses’ proactive vitality to success in dealing with challenging and uncertain circumstances, especially during and after pandemics.

**Purpose:**

This study was designed to develop a structural equation model to investigate the impact of resonant leadership on the proactive vitality management of nurses, with organizational compassion as a mediating variable.

**Methods:**

A cross-sectional survey with a convenience sampling technique was used on 520 nurses recruited from three public general hospitals in Alexandria, Egypt. Three instruments were used to collect the required data from January 2023 to June 2023, including the resonant leadership scale, nurses’ proactive vitality management scale, and organizational compassion scale.

**Results:**

The results of the structural equation model showed that the degree of fit for each index model was good, indicating all of the study variables, including resonant leadership and nurses’ proactive vitality management were directly affected by organizational compassion, confirming organizational compassion to be a mediating variable between resonant leadership and proactive vitality management.

**Conclusions:**

Organizational compassion is a mediating factor that enhances resonant leadership and stimulates nurses’ proactive vitality management. The research results suggest valuable protocols for health care organizations seeking to encourage nurses’ proactive vitality through effective emotional and resonant leadership in the context of organizational compassion.

## Introduction

The post–COVID-19 era is marked by disturbed individual and work lives as well as incredible vulnerability and tension, particularly for those working in the medical field. COVID-19 was a health care catastrophe, leading to an unprecedented impact on health care services and high rates of morbidity and mortality among both the public and health care providers. During the pandemic, health care providers experienced emotional exhaustion, which increased the incidence of medical errors, decreased empathy, reduced productivity, increased turnover rates, and decreased proactive vitality (Gupta et al., 2021). In this respect, health care organizations should adopt a more compassionate approach to operations by applying the core ethical principles of empathy toward employees, noticing changes in their behavior through continuous assessment, and responding to their suffering (Billings et al., 2021).

In addition, organizational leaders must be more emotionally responsive and provide compassionate support to their followers, which require resonant leadership. Salminen-Tuomaala and Seppälä (2022) found that resonant leaders rouse workers to decrease their dread of emerging from work misfortune and panic when losing hope, in turn promoting proactive vitality and productivity in nurses and improving patient outcomes (Blake, 2021, Wörtler et al., 2020). Thus, resonant leaders and organizational compassion may enhance proactive vitality in nurses and help them interpret the pandemic experience as a chance to make genuine change and discover new methods of working.

### Research Framework

**Figure 1:**
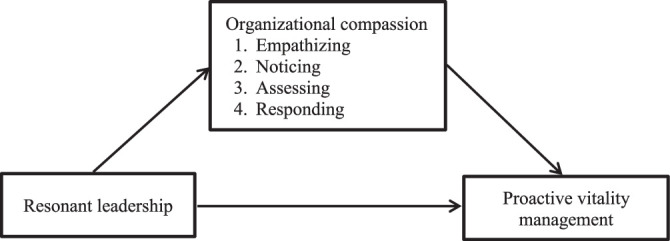
Conceptual Framework of the Study

The guiding framework for this research was social learning theory, which is increasingly cited as an essential component of sustainable natural resource management and the promotion of desirable behavioral change. This theory is based on the idea that we learn from our interactions with others in a social context and assumes that inspiration at work and positive relationships motivate employees to operate constructively (Bandura & Walters, 1977). Based on this perspective, resonant leadership is experienced by nurses when they are supported by organizational compassion, promoting their proactive vitality management. The conceptual framework for this study is shown in Figure 1.

### Resonant Leadership

COVID-19 challenged leaders to manage unanticipated changes and confronted them with unordinary circumstances that required them to be compassionate, empathetic, and passionate to form positive connections with their followers. Empathy and emotional intelligence are core characteristics of the resonant leadership style (Billings et al., 2021). Resonant leadership is the “behavior of leaders who demonstrate passionate insights, are sensitive to the feelings of the followers, utilize empathy, and oversee their feelings viably to construct solid, trusting connections, and create a climate of optimism that inspires commitment” (Squires et al., 2010).

A resonant leader focuses on the social and passionate requirements of their followers while being thoughtful, upbeat, and empathic, which leads to successful job outcomes. They support and involve their followers, inspiring them to achieve personal and organizational goals. Such leaders foster a dynamic work environment by instilling optimism, mindfulness, and compassion to encourage colleagues to combat fear arising from workplace tragedy and despair (Boyatzis et al., 2013). During difficult postpandemic periods, resonant leaders inspire their followers by displaying trust and nurturing, developing their staff (or team), and inspiring a perception of opportunities over barriers in their work. Thus, this type of leadership style can bring many benefits in the presence of effective organizational compassion to exhibit proactive vitality management among followers (Tiwari & Lenka, 2020).

### Organizational Compassion

Organizational compassion is “an interpersonal process including the noticing, feeling, sense-making, and acting that eases the enduring of another person” (Dutton et al., 2014). This conveys feelings of warmth, caring, and tenderness for colleagues or subordinates without the desire for any specific organizational advantage. Organizational compassion exists when individuals within a system perceive, feel, and respond to the suffering of others. It is a 4-step process: sense-making (evaluating or interpreting), noticing (being aware), empathizing (identifying with the suffering), and responding to that suffering (Eldor, 2018).

Through an organizational foundation of empathy, personnel view themselves as being favorably recognized through the willingness of others to help them leave or succeed in difficult working situations. Therefore, kindness is evident when one is receptive to and moved by the suffering of others, with the desire to alleviate that suffering, manifested through kindness, attention, and ordinary human decency toward the plight of others. It may be said that everyone’s role is to observe a colleague’s suffering; to provide sober assessments of that suffering, its causes, and the deservingness of the sufferer; and to determine the amount and type of support that is needed (Cameron, 2021).

The advantages of organizational compassion to individuals and organizations include faster posttrauma healing; improved confidence, pride, commitment, connection, and motivation in nurses; better organizational performance; and heightened nurse expectations of leader behaviors. Compassion lessens the effects of job-related stress and tensions in health care, contributes positively to rest quality and subjective well-being among medical attendants, and improves proactive vitality (Zhang et al., 2018).

### Proactive Vitality Management

The International Council of Nurses (ICN) issued the code of nursing ethics, which highlights that nurses bear the main responsibility for enhancing their professional development through continuous education. This requires nurses to take the initiative, be vigilant, and be proactive. However, COVID-19 significantly changed the landscape of the health care workplace, making it more stressful and leading to frontline staff members being ignored and vulnerable to intimidating and violent behaviors. This situation has weakened the proactive vigor of nurses and caused them to feel burned out and ethically wounded in addition to being physically and emotionally exhausted (ICN, 2021). Op den Kamp et al. (2018) explained proactivity as a “self-initiated and future-oriented activity that points to altering and progressing the circumstances or one.”

Proactive vitality management is “a personal, goal-oriented behavior directed to managing physical and mental power to enhance the optimal functioning at work” (Pap et al., 2022). It permits nurses to take charge of their diffuse emotional, affective, and cognitive resources to enhance their performance and well-being. When using proactive vitality management, individuals change their perspectives of themselves rather than of their workplace to realize a diverse future (Op den Kamp et al., 2018). Health personnel (such as nurses and doctors) deal routinely with shifting environments and are more prone to fatigue. Over time, they may become less persuaded because of their plan (night shifts, extra time, etc.). Proactive vitality management helps personnel avoid fatigue and hostile situations to provide better patient care. Therefore, the proactive nature of nurses is now considered crucial to meeting and exceeding business goals (Parker et al., 2010).

### Significance of This Study

The impact of the COVID-19 pandemic on the physical and mental wellness of health care workers, especially those involved in providing direct patient care as nurses, has been extensively studied. The results show that nurses faced numerous hardships during the pandemic, including moral distress, exhaustion, burnout, loss of vigor, and reduced ability to contribute significantly to their work, resulting in poor performance and productivity. However, health care organizations rely on proactive health care providers to rapidly adapt to demanding contexts and respond productively to pandemic-related crises (Ali Awad & Al-anwer Ashour, 2022; Ali Awad & Mohamed El Sayed, 2023; Nimako & Basatan, 2022).

It is important to recognize the efforts made by managers of health care organizations to foster organizational compassion in the health care setting and develop resonant leadership (based on the emotional intelligence and empathy of leaders) to improve proactive vitality management in nurses. Although organizational compassion, resonant leadership, and proactive vitality management have all been previously studied, no relationship model for nurses that triangulates these three ideas in the post–COVID-19 context has been proposed. Therefore, there is a significant need to address this relationship to close the current gap in the literature by examining whether organizational compassion acts as mediation between resonant leadership and the proactive vitality management of nurses. As COVID-19 will likely not be the only pandemic the world will face in the coming years, health care organizations must create a foundational support system to enhance proactive vitality in nurses through a resonant leadership style that fosters organizational compassion.

### Research Aims and Hypothesis

The aim of this study was to develop a structured equation model to examine the effect of resonant leadership as an independent variable on the dependent variable of proactive vitality management in nurses through the mediator of organizational compassion. The research question and hypothesis are presented as follows (see also Figure 1):

How does organizational compassion mediate the relationship between resonant leadership and proactive vitality management in nurses?

Alternative H1: The influence of resonant leadership on proactive vitality management in nurses is mediated positively and significantly by organizational compassion.

## Methods

### Design and Setting

A cross-sectional design was used in this study to clarify how variables relate to one another over a predetermined period, and a model was constructed to analyze how resonant leadership affects nurses’ proactive vitality management when post–COVID-19 organizational compassion acts as a mediating factor. This study was conducted at three urban public general hospitals in Alexandria City, Egypt. The researchers chose four departments from each hospital using a simple random method. These hospitals are the largest general hospitals in Alexandria, with the largest health care teams. Also, they receive patients from a wide range of governorates in Egypt. They are multispecialty hospitals and provide comprehensive health care services such as inpatient, outpatient, intensive care and critical care, emergency, radiologic, laboratory, and physiotherapy services. Moreover, they provide teaching and clinical training services for medical and nursing students and provided services during the pandemic.

### Participants

The patients recruited for this study comprised a convenient, nonprobability sample of 520 bedside nurses who all had more than three months of experience and volunteered to participate. Nurse interns and ill nurses were excluded participation. The questionnaire was distributed to the participants from early January through June 2023. Returned questionnaires were vetted, and those with any one of the following issues were disqualified: from the same hospital and showed similar responses, answers followed a pattern (e.g., option “3” was ticked for all questions), more than one answer was provided for a single-choice question, and leaving more than 5% of questionnaire items unanswered. Mean imputation was employed for questionnaires to account for missing answers on the included questionnaires. A total of 470 surveys were deemed valid after filtering and were included in the analysis.

### Data Collection

Three instruments, including the resonant leadership scale, proactive vitality management scale, and organizational compassion scale, were used to gather study data and test the hypothesis.

### Instruments

#### Resonant Leadership Scale

The RLS, originally proposed by Cummings et al. (2010), uses 10 statements to measure resonant leadership in a leader from the perspective of nurses. Example items include: “My leader effectively resolves conflicts that arise” and “My leader engages me in working toward a shared vision.” A 5-point Likert Scale ranging from (1) *strongly disagree* to (5) *strongly agree* is used for item scoring, with a total possible scale score ranging from 10 to 50. Mean percentage scores of >66.6%, 66.6%–33.3%, and <33.3%, respectively, indicate high, moderate, and low levels of resonant leadership.

#### Proactive Vitality Management Scale

The Proactive Vitality Management (PVM) Scale, developed by Op den Kamp et al. (2018) and validated by Bălăceanu et al., (2022), uses 8 items to measure employee energy (proactive vitality) at work. Items are scored using a 5-point Likert Scale ranging from (1) *strongly disagree* to (5) *strongly agree*, with a total possible scale score ranging from 8 to 40. Mean percentage scores of >66.6%, 66.6%–33.3%, and <33.3%, respectively, indicate high, moderate, and low levels of proactive vitality in respondents.

#### Organizational Compassion Scale

The OCS, developed by Simpson and Farr-Wharton (2017), was used in this study to assess the ability of participants to notice, empathize, assess, and respond to suffering. This scale consists of 20 items divided into four dimensions namely, Noticing (6 items), Empathizing (5 items), Assessing (3 items), and Responding (6 statements). Responses are measured on a 5-point Likert Scale ranging from (1) *strongly disagree* to (5) *strongly agree*, with a total possible scale score ranging from 20 to 100. Mean percentage scores of >66.6%, 66.6%–33.3%, and <33.3%, respectively, indicate high, moderate, and low levels of organizational compassion.

#### Demographic Information Sheet

A sociodemographic information sheet designed by the researchers was used to gather data on participant age, sex, educational level, and years of work experience.

### Procedures

After an official permission was obtained from Alexandria University’s Faculty of Nursing Research Ethics Committee (IBR00013620), the researchers obtained written consent from the regulatory specialist to collect the necessary data. To assess the clarity and suitability of the tools and determine the amount of time required to complete the questionnaires, a pilot study for the questionnaires was carried out on 10% of the study sample (*n* = 50) to check and ensure the clarity of the questionnaires, identify obstacles and problems that may be encountered during data collection, and estimate the time needed to complete the questionnaires. Before data collection, informed consent to participate in the study was obtained from the participants. The privacy and confidentiality of all information collected were ensured. The researcher hand-delivered the questionnaires after meeting with each participant to explain the purpose of the study, provide any needed clarification, and confirm the voluntary nature of participation. About 30 minutes were required to complete the questionnaires.

### Validity and Reliability

After the study tools were translated into Arabic and adjusted for Egyptian culture, a board of seven academic experts evaluated their content validity. Also, the study tools were tested for internal reliability using Cronbach’s alpha correlation coefficient and found to be reliable, with α = .950, .886, and .940, respectively, for resonant leadership, proactive management vitality, and organizational compassion, at a reliability level of *p* = .05.

### Data Analysis

The data were examined using Analysis of Moment Structures (AMOS) Ver. 23. AMOS is an IBM SPSS statistics module designed to analyze covariance structure models, including structural equation modeling, path analysis, and confirmatory factor analysis (CFA). In addition, the SPSS is a suite of software programs designed to analyze scientific data related to the social sciences. Frequency, percentage, mean percent, and *SD* were used in this study to analyze demographic statistics, resonant leadership, PVM, and organizational compassion. Pearson’s correlations were used to determine the relationships between resonant leadership, proactive vitality management, and organizational compassion. Cronbach’s alpha was used to evaluate the dependability of the tools. The mediation effect was examined using structural equation modeling and AMOS Ver. 23. The suitability of the model was determined by gauging the suitability of each latent variable structure. Indicators used to confirm the structure of the model’s fit indices included the χ^2^ values, the Root Mean Square Error of Approximation (RMSEA), Normed Fit Index (NFI), Comparative Fit Index (CFI), and Relative Fit Index (RFI). A significant χ^2^ score implies the model and the data fit well. RFI, NFI, and CFI values >.90 denote a good match, while RMSEA values <.08 imply an acceptable fit and those at the .05 level imply the path coefficients to be significant.

### Control Variables

Demographic variables, including age, sex, educational level, and years of work experience, were controlled in this study, as these characteristics may influence the dependent variable.

## Results

As shown in Table 1, the participants earned a low mean percentage score for overall organizational compassion (33.54, *SD* = 4.87), which was illustrated in all aspects in the following numerical order: assessing (32.64, *SD* = 5.93), responding (30.92, *SD* = 4.79), noticing (30.29, *SD* = 4.63), and empathizing (29.86, *SD* = 4.77). In addition, the participants reported a moderate mean percentage score for resonant leadership (40.49, *SD* = 7.0) and proactive vitality (35.86, *SD* = 5.35).

**Table 1:** Mean Percentage Scores of Perceptions on Organizational Compassion and Its Related Aspects, Resonant Leadership, and Proactive Vitality Management

Study Variable	Minimum–Maximum	*M* (*SD*)
Noticing	11.33–40.67	30.29 (4.63)
Empathizing	11.00–40.00	29.86 (4.77)
Assessing	9.67–43.33	32.64 (5.93)
Responding	15.00–41.67	30.92 (4.79)
Overall organizational compassion	32.00–92.00	33.54 (4.87)
Resonant leadership	17.00–52.00	40.49 (7.0)
Proactive vitality	14.50–45.50	35.86 (5.35)

Proactive vitality and resonant leadership showed a statistically significant and positive relationship, with *p <* .01, as shown in Table 2. Also, statistically significant and positive associations were identified between resonant leadership and organizational compassion and its subdimensions of noticing, empathizing, assessing, and responding (all *p* < .01). Furthermore, statistically significant and positive associations were identified between organizational compassion and proactive vitality and their related constructs of noticing, empathizing, evaluating, and responding (all *p* < .001).

**Table 2:** Correlational Matrix Among Resonant Leadership, Proactive Vitality Management, and Organizational Compassion

Study Variable	Resonant Leadership	Proactive Vitality	Noticing	Empathizing	Assessing	Responding	Organizational Compassion
Resonant leadership							
*r*	—	.636^*^	.442^*^	.473^*^	.363^*^	.528^*^	.606^*^
*p*	—	<.001	<.001	<.001	<.001	<.001	<.001
Proactive Vitality							
*r*	.636^*^	—	.527^*^	.579^*^	.444^*^	.518^*^	.677^*^
*p*	<.001	—	<.001	<.001	<.001	<.001	<.001
Noticing							
*r*	.442^*^	.527^*^	—	.505^*^	.332^*^	.491^*^	.790^*^
*p*	<.001	<.001	—	<.001	<.001	<.001	<.001
Empathizing							
*r*	.473^*^	.579^*^	.505^*^	—	.351^*^	.524^*^	.774^*^
*p*	<.001	<.001	<.001	—	<.001	<.001	<.001
Assessing							
*r*	.363^*^	.444^*^	.332^*^	.351^*^	—	.317^*^	.521^*^
*p*	<.001	<.001	<.001	<.001	—	<.001	<.001
Responding							
*r*	.528^*^	.518^*^	.491^*^	.524^*^	.317^*^	—	.808^*^
*p*	<.001	<.001	<.001	<.001	<.001	—	<.001

^*^ Correlation is significant at the .01 level (2-tailed).

**Figure 2:**
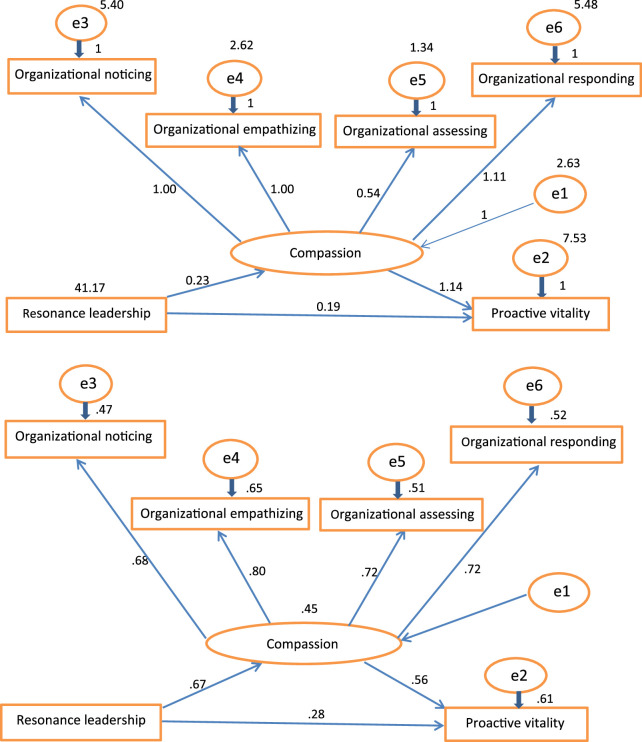
Path Analysis for Direct and Indirect Effect of Resonant Leadership on Nurses’ Proactive Vitality Management Mediated by Organizational Compassion

As shown in Figure 2 and Table 3, testing of the structured models was done to confirm the research hypothesis. According to the test results, goodness of fit index values included RMSEA = .033, RMR = .273, GFI = .98, NFI = .98, NNFI = 1.02, RFI = .97, and Normed χ^2^ = 10.178 with a *p*-value of .001. Thus, the overall fit of the structural model was good and in line with the research data. The results generated by the model indicate resonant leadership affects organizational compassion significantly, with an estimated β of 0.228 and a coefficient of regression CR of 9.473 (*p* < .001). Also, resonant leadership was shown to have a significant effect on proactive vitality management, with an estimated β of 0.192 and a coefficient of regression CR of 4.464 (*p* < .001). Furthermore, this model illustrates the significant effect of organizational compassion on proactive vitality with an estimated β of 1.136 and a coefficient of regression CR of 7.200 (*p* < .001). Thus, based on the results of this model, organizational compassion significantly and positively mediates the relationship between resonant leadership and proactive vitality.

**Table 3:** Path Analysis of the Direct and Indirect Effects of Resonant Leadership on Proactive Vitality Management Mediated by Organizational Compassion

Outcomes	Predictors		Estimate	*SE*	CR	*p*
Organizational compassion	←	Resonance Leadership	0.228	0.024	9.473	<.001
Proactive vitality	←	Resonance Leadership	0.192	0.043	4.464	<.001
Proactive vitality	←	Compassion	1.136	0.158	7.200	<.001
Organizational noticing	←	Compassion	1.000	—	—	<.001
Organizational empathizing	←	Compassion	1.003	0.091	10.979	<.001
Organizational assessing	←	Compassion	0.543	0.054	9.996	<.001
Organizational responding	←	Compassion	1.111	0.111	10.041	<.001

*Note.* CR = coefficient of regression.

## Discussion

In our current era, in which nurses working in health care organizations face many challenges that may cause them to feel discouraged, compassion is imperative to helping nurses overcome harmful situations. Moreover, organizations may empower organizational compassion by fortifying shared convictions and values and fostering social aptitudes, which encourage noticing, feeling, and responding to the pain of others (Dutton et al., 2014). Also, organizations may foster healing environments in which every member experiencing suffering is able to sense the support of their organization. Systemic organizational elements such as beliefs, practices, and routines that support a group’s ability to perceive, feel, and respond to suffering are necessary to spread compassion in companies (Kanov et al., 2004).

In this study, the participants perceived organizational compassion to be inadequate because their organizations lack systems for dealing with their suffering and pain. In addition, they reported their organizations did not attempt to assist or respond to relieve their suffering or provide structure or formal programs to enhance their emotional recovery. This finding is similar to Hamza (2021), who found a low level of organizational compassion, but contradicted Muzee et al. (2021), who found a moderate level of organizational compassion, and Guinot et al. (2020) and Guo and Zhu (2022), who found high levels of organizational compassion.

Ali and Kashif (2020) concluded that resonant leaders can help emotionally heal followers involved in highly overworked health care jobs, promoting pleasant emotions. However, the findings of this study showed the participants rated their leaders as having only a slightly moderate level of resonant leadership, and identified their leaders as facing difficulties in trying to establish strong emotional bonds with their employees, spread hope among organizational staff, and use their emotional intelligence and that they dealt with staff with inadequate empathy toward their feeling and needs, thus hindering them from establishing stronger emotional ties with their followers. This result is similar to Bawafaa et al. (2015); Laschinger et al. (2014); and Reynolds et al. (2022) who all concluded that nurses view their leaders as having a mean moderate level of resonant leadership, but contradicted Gaan and Shin (2022) and Parr et al. (2021) who found subjects identified their managers as exhibiting high levels of resonant leadership.

In today’s energetic working environments, proactive behavior in individuals is crucial to their work execution. Work proactivity means “demonstrating activity to advance existing circumstances or creating new conditions; it includes mobilizing the status quo instead of latently adjusting to show conditions” (Bakker et al., 2020). However, with regard to the perceived importance of proactive behavior to nurses and the health care organization, the participants in this study reported a slightly moderate level of proactive vitality management. This result may be explained as the participants feeling a lack of energy and power and becoming demotivated to accomplish their duties on time. Furthermore, this may be secondary to the moderate level of resonant leadership and low level of organizational compassion, which lead to the ineffective generation of trust, connection, and positive emotions that affect nurses’ creativity and proactive vitality.

The findings of Tummers et al. (2015) study contradict the current results, in which employees were shown to have low level of proactive vitality management. Moreover, proactivity and invigorating feelings of essentiality were found by Binyamin and Brender-Ilan (2018) to be positively correlated. However, the claims in Op den Kamp et al. (2018) and Op den Kamp et al. (2023) that nurses assessed high proactive vitality management contrast with this finding.

This study is ground-breaking in terms of demonstrating the importance of resonant leadership in predicting proactive vitality management through the mediating function of organizational compassion in nurses during the transition out of the COVID-19 pandemic. Given this outcome, the finding that resonant leadership, proactive vitality, and organizational compassion shared statistically significant and positive correlations was unexpected. This model also demonstrates the role of organizational compassion in mediating the relationship between resonant leadership and proactive vitality. Worline et al. (2017) provide evidence to support this, outlining how compassion at work increases collective capacities such as creativity, invention, and learning, which fuels competitive advantage. In addition, Guinot et al. (2020) and Guo and Zhu (2022) provided evidence of the mediating role of organizational compassion. Furthermore, Ali and Kashif (2020), Bawafaa et al. (2015), Binyamin and Brender-Ilan (2018), and Vogus et al. (2021) found evidence in support of the mediating effect of organizational compassion and clarified a significantly positive relationship between organizational compassion and resonant leadership. Also, leaders who demonstrate a resonant style can comfort their followers emotionally in the presence of effective organizational compassion, and subsequently stimulate employees to exhibit PVM. In this context, studies with similar findings may have focused on examining psychological phenomena that all health care providers have faced both during and after the pandemic. Moreover, these phenomena are not greatly influenced by cultural or contextual factors. However, other studies have reported different conclusions, which may be explained by the application of methodologies including settings, sample sizes, and research designs, and the researchers may have adopted different theoretical frameworks to guide research hypotheses. All these factors may have affected data analysis during the study period, resulting in different conclusions.

### Conclusions and Recommendations

This study explored the significant and positive impact of applying ethical principles of organizational compassion such as empathy, making sense, and responding to nurses’ suffering during and after the recent COVID-19 pandemic. Organizational compassion is a mediating factor in enhancing resonant leadership that improves PVM in nurses. Thus, this research provides procedures for health care institutions to strengthen nurses’ proactive vitality management by practicing a resonant leadership style and promoting organizational compassion in the workplace. This will support and empower nurses during any pandemic or crisis and achieve effective work outcomes.

### Relevance to Clinical Practice

The COVID-19 pandemic highlighted the value of sound leadership. To support PVM in nurses and pursue their development of resonant leadership and organizational compassion, this inquiry offers some urgent commonsense recommendations for nurse managers and health care executives. The findings of this study provide organizations with a roadmap of actions that may be communicated to representatives to improve their PVM by showcasing the success of ground-breaking organizational compassion mediation between resonant leadership and proactive vitality. We equip managers with the resources needed to better train their staff members on PVM so they may perform their best at work. Hospital management should ensure organizational compassion, resonant leadership, and PVM by forging bonds with supporters, inspiring optimism, and developing a culture of support and positivity at work, which requires managing novel and troublesome circumstances.

### Limitations

The findings of this study add considerably to the existing research by building a structure equation model among study variables that have not been discussed previously in the literature. However, some limitations were encountered in this study. As the participants were drawn from a specific setting for convenience; the generalizability of the outcomes is restricted. Also, the findings are drawn from self-narrated data, which may be susceptible to response bias and subjectivity. Finally, the data were collected from 3 different hospitals, which may introduce variability.
